# The Lumbar Lordosis in Males and Females, Revisited

**DOI:** 10.1371/journal.pone.0133685

**Published:** 2015-08-24

**Authors:** Ori Hay, Gali Dar, Janan Abbas, Dan Stein, Hila May, Youssef Masharawi, Nathan Peled, Israel Hershkovitz

**Affiliations:** 1 Department of Anatomy and Anthropology, Sackler Faculty of Medicine, Tel Aviv University, Tel Aviv, Israel; 2 Department of Physical Therapy, Faculty of Social Welfare and Health Sciences, University of Haifa, Haifa, Israel; 3 Zefat Academic College, Zefat, Israel; 4 Department of Physiotherapy, School of Health Professions, Tel-Aviv University, Te-Aviv, Israel; 5 Department of Radiology, Carmel Medical Center, Haifa, Israel; University of Delaware, UNITED STATES

## Abstract

**Background:**

Whether differences exist in male and female lumbar lordosis has been debated by researchers who are divided as to the nature of variations in the spinal curve, their origin, reasoning, and implications from a morphological, functional and evolutionary perspective. Evaluation of the spinal curvature is constructive in understanding the evolution of the spine, as well as its pathology, planning of surgical procedures, monitoring its progression and treatment of spinal deformities. The aim of the current study was to revisit the nature of lumbar curve in males and females.

**Methods:**

Our new automated method uses CT imaging of the spine to measure lumbar curvature in males and females. The curves extracted from 158 individuals were based on the spinal canal, thus avoiding traditional pitfalls of using bone features for curve estimation. The model analysis was carried out on the entire curve, whereby both local and global descriptors were examined in a single framework. Six parameters were calculated: segment length, curve length, curvedness, lordosis peak location, lordosis cranial peak height, and lordosis caudal peak height.

**Principal Findings:**

Compared to males, the female spine manifested a statistically significant greater curvature, a caudally located lordotic peak, and greater cranial peak height. As caudal peak height is similar for males and females, the illusion of deeper lordosis among females is due partially to the fact that the upper part of the female lumbar curve is positioned more dorsally (more backwardly inclined).

**Conclusions:**

Males and females manifest different lumbar curve shape, yet similar amount of inward curving (lordosis). The morphological characteristics of the female spine were probably developed to reduce stress on the vertebral elements during pregnancy and nursing.

## Introduction

The ambiguity of dissimilarity between male and female spinal configurations has intrigued many researchers over the past few decades (for review see Been and Kalichman [1][1]). This vagueness relates not only to uncertainty as to the existence of a difference in posture, but also to questions regarding the nature of variations, their origin, reasoning, and implications from morphological and functional points of view. There are numerous publications about the human lumbar lordosis-about half of the studies found no statistical difference between males and females and the other half found a small difference [2]. To add to the ambiguity of the differences between male and female posture, the very definition and measurement of posture poses complex challenges.

**Fig 1:**
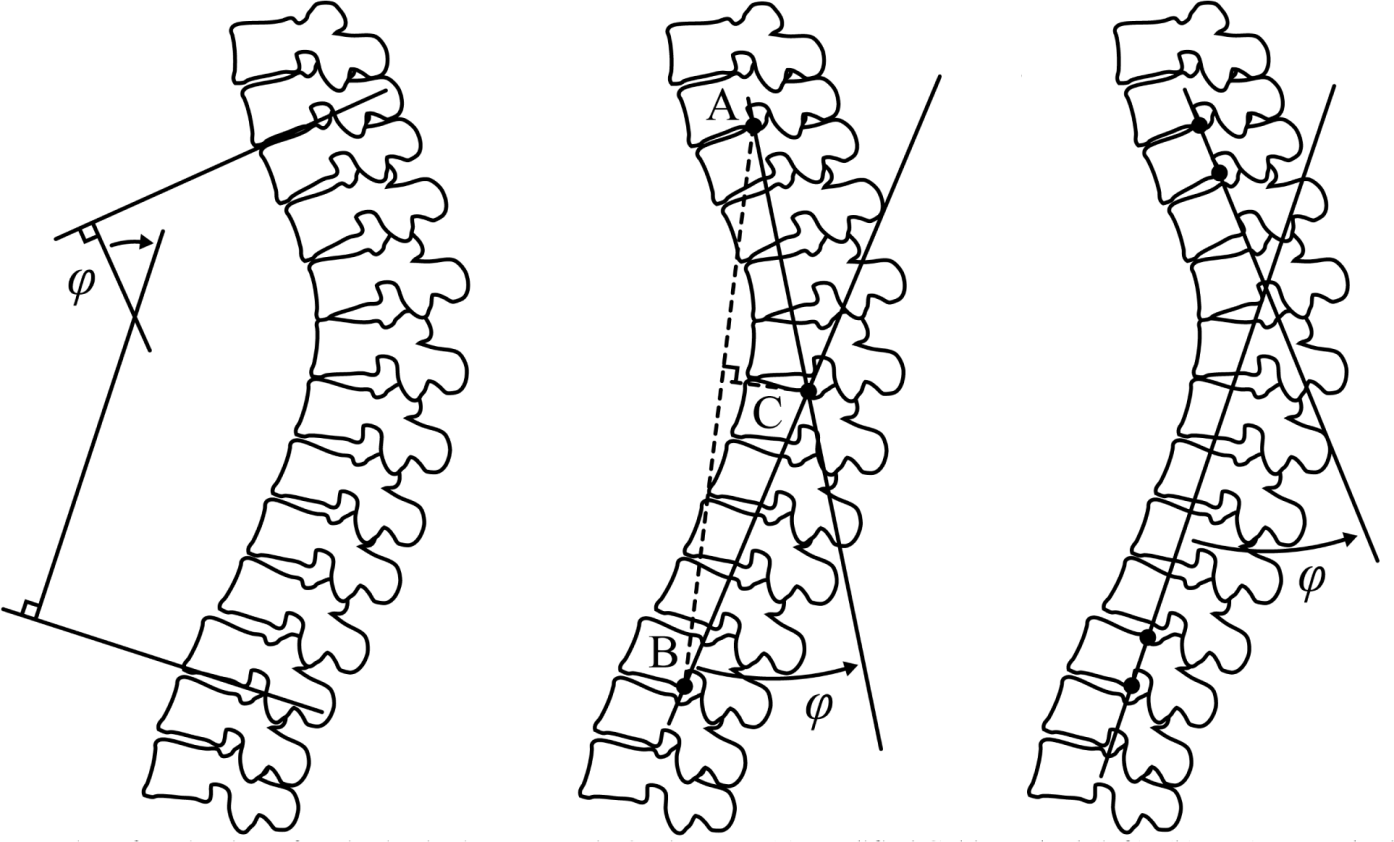
Examples of spinal curvature evaluation methods. Methods of evaluating the sagittal spinal curvature in 2D images: (a) Modified Cobb method (left); (b) TRALL method (middle); (c) Posterior tangents (right).

Creating simple measures for spine configuration is a challenging task. Most measurements involve only small aspects of the spinal configuration and are two-dimensional (2-D) in nature, therefore failing to capture the large variety of spinal curve shapes. Most of these measurement methods lack the 3-D aspects of the spine curve shape. For example, measuring the lumbar lordosis via imaging [3] such as the Cobb angle [4] [4], TRALL or tangents methods [5] (Fig 1) can identify only large differences, but are insensitive to minor and local deviations. Indeed, some of the available methods can also be applied locally, i. e., to a more specific region of the curve (e.g., measuring the angle for neighboring vertebrae), but these measurements fail to depict more comprehensive aspects of the spinal posture (e.g., the geometrical shape of the curve in 3-D, simultaneously describing local and global shape characteristics). This methodological shortcoming explains why there is uncertainty as to posture differences between males and females.

### Previous work

#### Lumbar curvature evaluation

Evaluating the curvature of the spine is crucial in diagnosing pathological spinal deformities, formulating orthopedic surgical procedures, monitoring progression and treatment of spinal deformities and determining reference values in normal and pathological conditions. However, the human capability (i.e., naked-eye observation) to quantitatively evaluate spinal configuration from medical images is limited partly due to similar characteristics of normal and pathological conditions, and the natural biological variability in spinal architecture (e.g., thoracic T4-T12 kyphosis angle ranging from 0° to 69° with a mean value of 40° and lumbar L1-L5 lordosis angle ranging from -69° to -13.6° with a mean value of -43° [2]). Technical limitations (such as image noise), imaging technique characteristics, and available image analysis tools also represent a major source of variability that may conceal the actual geometrical relationship between anatomical structures, thus introduce evaluation errors. Although 2-D images are still widely used in clinical examinations, advances in medical technology have led to the development of new 3-D imaging techniques that have become important clinical tools in modern health care. Nowadays, 2-D radiographic (x-ray) images are frequently replaced by 3-D images obtained from computed tomography (CT) and magnetic resonance (MRI) imaging. To overcome the difficulties of quantitative spinal curvature evaluation, numerous methods have been developed over the years (Fig 1).

The Cobb method [4] is the more common method and one of the first used to evaluate spinal curvature. Nevertheless, this method has several reported limitations such as the inability to identify local curvature changes [6], poor accuracy and large variability [7, 8]. Several alternative methods have been proposed, for example the Ishihara Index, the Polynomial Angle, the TRALL Method, and Posterior Tangents. These methods were originally designed for plain radiographs, but were later adapted to 3-D imaging. To date, there is no accurate, widely accepted method for measuring spinal curvature specifically designed for 3-D images [3].

#### Differences between males and females

Many studies have debated whether differences exist between male and female spinal architecture. Several sagittal x-ray studies measuring lordosis differences have been conducted. Vialle et al. [2] observed a difference in lumbar lordosis in males and females (larger lordosis in females as measured by the Cobb angle). Similarly, based on a 3-D electromechanical digitizer to derive curvature angles for the region of the spine between T12-L1 and S2, Norton et al. [9] found that the lordosis angle (calculated using the ratio between the lordosis depth and length) was 13.2° larger for women than for men. On the other hand, a study conducted using the Cobb angle measurement on plain radiographs (x-rays taken of individuals in a standing position) of an asymptomatic Greek population, demonstrated that thoracic kyphosis and lumbar lordosis (T12-S1, L1-L5) were not sex-related [10].

A recent study conducted on 60 healthy males and females using innovative upright low-dose digital biplanar x-rays and three-dimensional analysis [11] also failed to reveal a significant difference in lordosis between males and females. The study also examined the sagittal spinal inclination defined as the angle between a vertical and best-fit straight line passing through the centroids of the vertebrae and found that the thoracic and thoracolumbar vertebrae were more dorsally (backwardly) inclined in women than in men.

The aim of the current study is to illustrate the spinal lumbar curve in males and females, compare them, and determine references for normal and pathological conditions. Our working hypothesis is that males and females will exhibit a similar lumbar curve shape.

## Materials and Methods

### Ethics statement

Our study was approved by the Human Research Committee of the Carmel Medical Center. Written or oral informed consent was given. Verbal consent (approval by phone) was accepted by the Ethics Committee per our request.

This is a retrospective study based on CT scans of outpatients retrieved from the hospital’s database. Nearly all participants were discharged from the hospital shortly after the scan. Initially, recruiting was carried out on-site with written consent, however, this process proved stressful to the patients and time-consuming. Per our request, the Ethics Committee approved the use of previous scans together with verbal informed consent.

Verbal consent was given by phone with each call documented into the study's data files. A list of potential participants (those who had undergone an abdominal CT) was retrieved from the hospital records. Each potential examinee was asked to authorize (or decline) the inclusion of their scan into the study. Those agreeing to participate in the study were also asked to fill out a short questionnaire. Records of each call were kept.

### Clinical data

This research is based on abdominal CT images (covering the entire lumbar spine and sacrum) performed when the patient was lying on the scanning bed in a standard supine position. The study sample included 158 patients; 81 males and 77 females, chosen from 421 individuals. Exclusion criteria were: past or present back pain, presence of spinal diseases (e.g., spondyloarthropathy, DISH, etc.), spinal configuration anomalies (e.g., scoliosis: Cobb angle >10° [12][12]), history of abdominal trauma or surgery, metabolic diseases, cancer, or muscoloskeletal diseases, refusal to participate in the study or fill out the questionnaire, or failure of the automated algorithm to perform properly for their CT scans (e.g., due to low resolution, etc.).

Most participants had been referred for a CT scan following abdominal problems. The average age of the study sample was 42.2±14.9 years. Images were obtained from a Philips scanner (Philips brilliance 64) and processed at a Philips work station. Voxel size in-plane ranged from 0.5–1.2mm; slice thickness was 0.9–3mm. Each image plane had 512 x 512 voxels with varying number of slices (250–700).

### Model comparison

In order to investigate the differences in spine configuration between males and females, we devised a new automated method for assessing the curvature of the spine in general, and the lumbar spine region in particular. The new automated method uses CT imaging of the spine to obtain a 3-D curvature description of the spine. Using a large number of sample scans and the appropriate extracted curves from each individual, we were able to create a model curve of the spinal canal for each population that could then be compared with each other. The term “Model” in our study is therefore refers to the average spinal canal curve of a given sample and its statistical parameters (e.g. mean curve and the SD of the curve).

The lumbar curves were based on the spinal canal which we regarded as a 3-D object. They were not based on features of the vertebral bodies, thus avoiding the pitfalls of using skeletal landmarks for curve estimation. For example, normal spine configuration with the presence of osteophytes at the cranial dorsal margin of L1 may greatly hamper the degree of the lordosis Cobb angle (L1-S1). The canal centerline curve, on the other hand, is unaffected by growing osteophytes along the discal surfaces rim. Moreover, local bony changes within the canal may only result in negligible localized changes of the curve that have insignificant impact on the analysis of the general curve. The model analysis was based on the entire curve, therefore both local and global descriptors were examined in a single framework.

**Fig 2:**
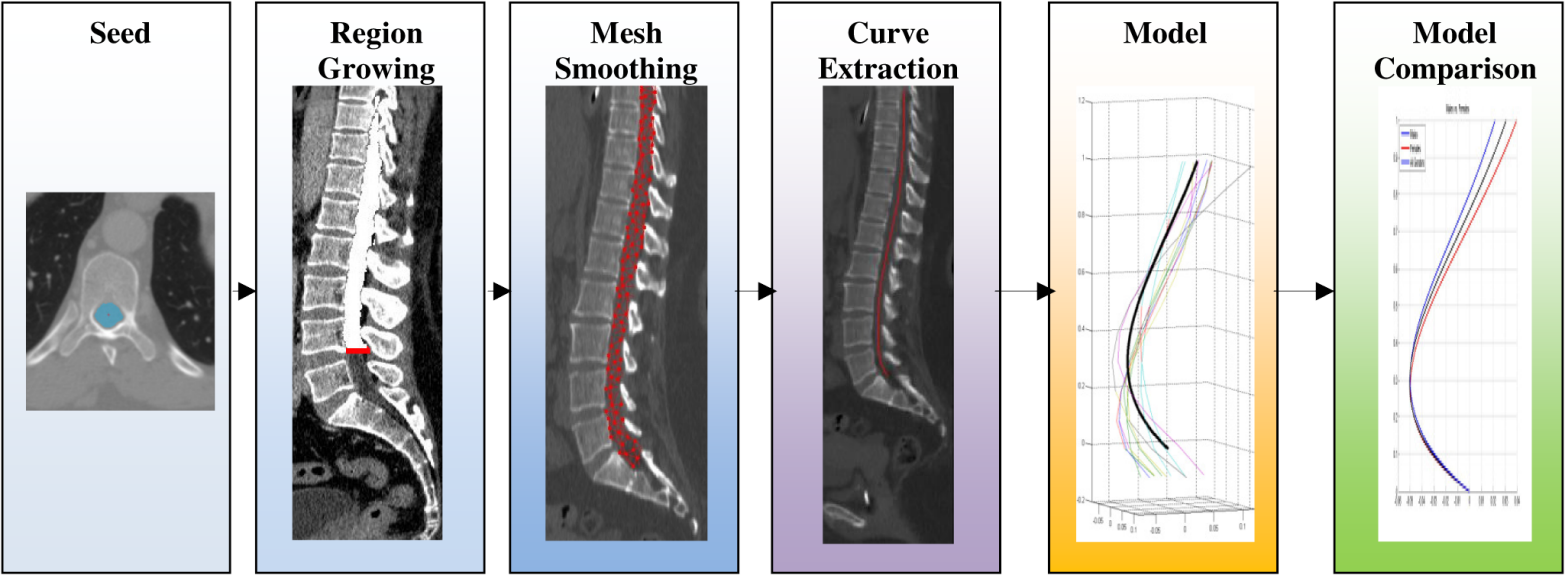
The spinal curve assessment framework. The spinal curve assessment framework steps (shown from left to right): the first 3 steps (on the left, colored blue) are spinal canal segmentation steps—seed point, fast growing morphological region and active object segmentation. The fourth step (middle, purple) is curve extraction. The next curve model (5th step from the left, yellow) is created and finally model comparison (right, green).

The spine assessment framework algorithmic steps were are presented in Fig 2: (1) segmentation of the spinal canal—a segmentation algorithm employing a two-step coarse to fine segmentation extracting the spinal canal; (2) extraction of the lumbar curve—after segmentation of the spinal canal, the lumbar curve was found using a minimal path algorithm applied to the segmented spinal canal volume; (3) curve modeling—based on curves extracted from healthy individuals; and (4) a model comparison of the two populations and assessment of the differences. Implementation was carried out in C++ using the ITK library, an algorithm developed by Ori Hay and Dan Stein.

#### Segmentation of the spinal canal

The spinal canal can be roughly regarded as a tube-shaped object with sharp edges (on the vertebrae), weak edges (on the intervertebral discs) and no edges (on the nerve roots), thus making segmentation of the canal a challenging task. Several techniques as to how to extract the canal have been suggested [13,14].

The canal segmentation algorithm is composed of two successive steps: initial segmentation carried out using a morphological region growing technique (applying morphological information–shape and size of candidates regions—to the selection to grow in the iterative process of region growing segmentation), then followed by a fine 3-D active surface segmentation [15]. This method allows a very fast (within 2–3 seconds for 400 images) initial segmentation followed by a fine and accurate adaptation of the segmentation to the canal shape.

#### Extraction of the lumbar curve

In our method, the spinal canal centerline defines the lumbar curve. The centerline extraction was carried out using a fast marching minimal path extraction technique [16] applied to the result of the spinal canal segmentation. After the spinal canal curve was established, the curve was scaled by the projected cranial-caudal axis distance between centerline points at the level of the inferior endplate of T12 to the superior endplate of S1, obtaining an independent measure curve (i.e., a curve independent of a patient’s height), and thus rotated so that the dorsal-ventral line was oriented on a common axis (Y axis). Scaling and rotating were used to establish an independent coordinate system for that particular patient’s spine curvature. The coordinates were defined relative to the scanned individual regardless of his position (patient coordinates) as follow: Y-axis = dorsal-ventral, X-axis = lateral-lateral, Z-axis = cranial-caudal.

#### Building model of the lumbar curve

**Fig 3:**
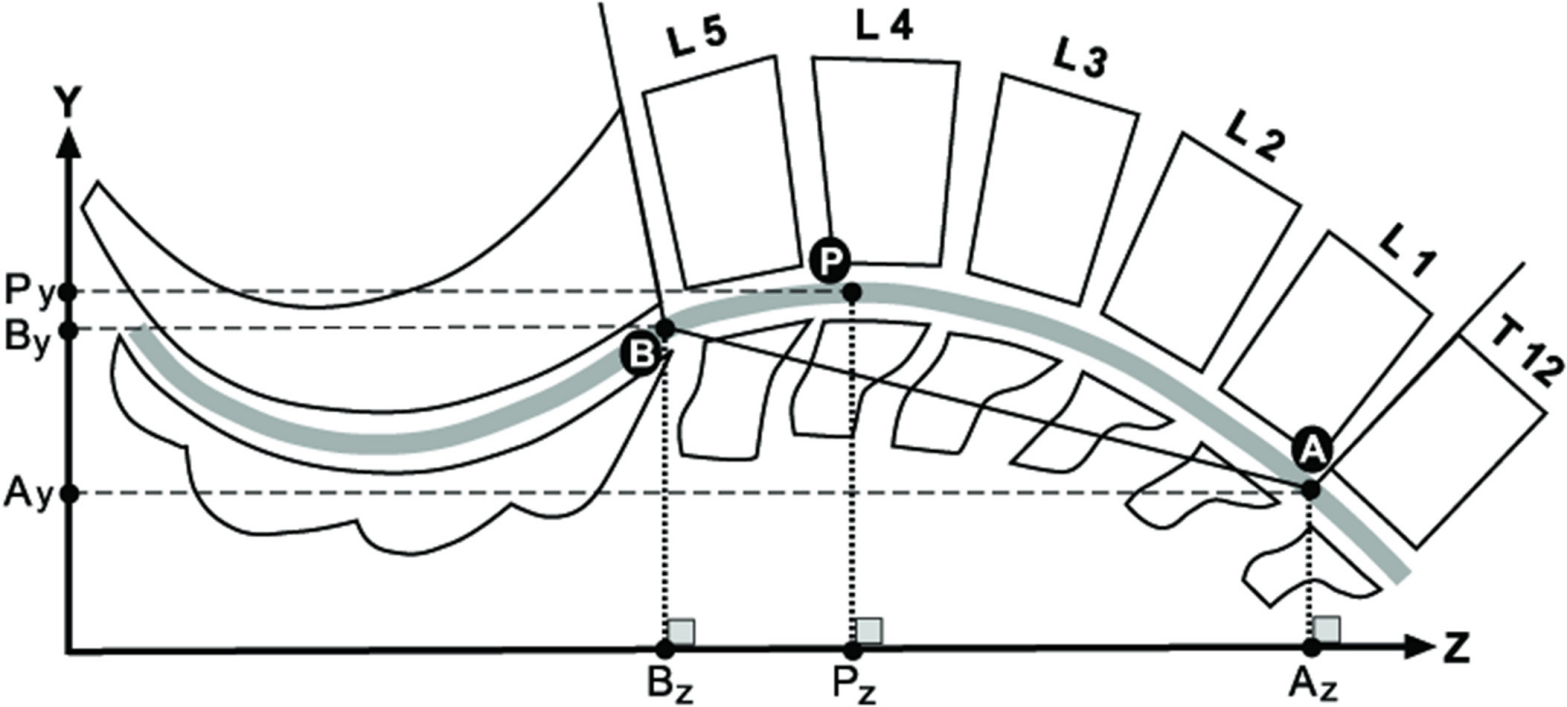
The spinal curve characteristics measurements. Schematic of the curve's characteristics measurements showing the vertebrae. The spinal canal centerline is presented as a wide gray line. The curve endings are marked as points A (cranial curve ending) and B (caudal curve ending), and the lordosis peak is marked by point P. The projections of each point on the Z and Y axes are also drawn as Az, Bz, Pz, and Ay, By, Py, respectively.

**Fig 4:**
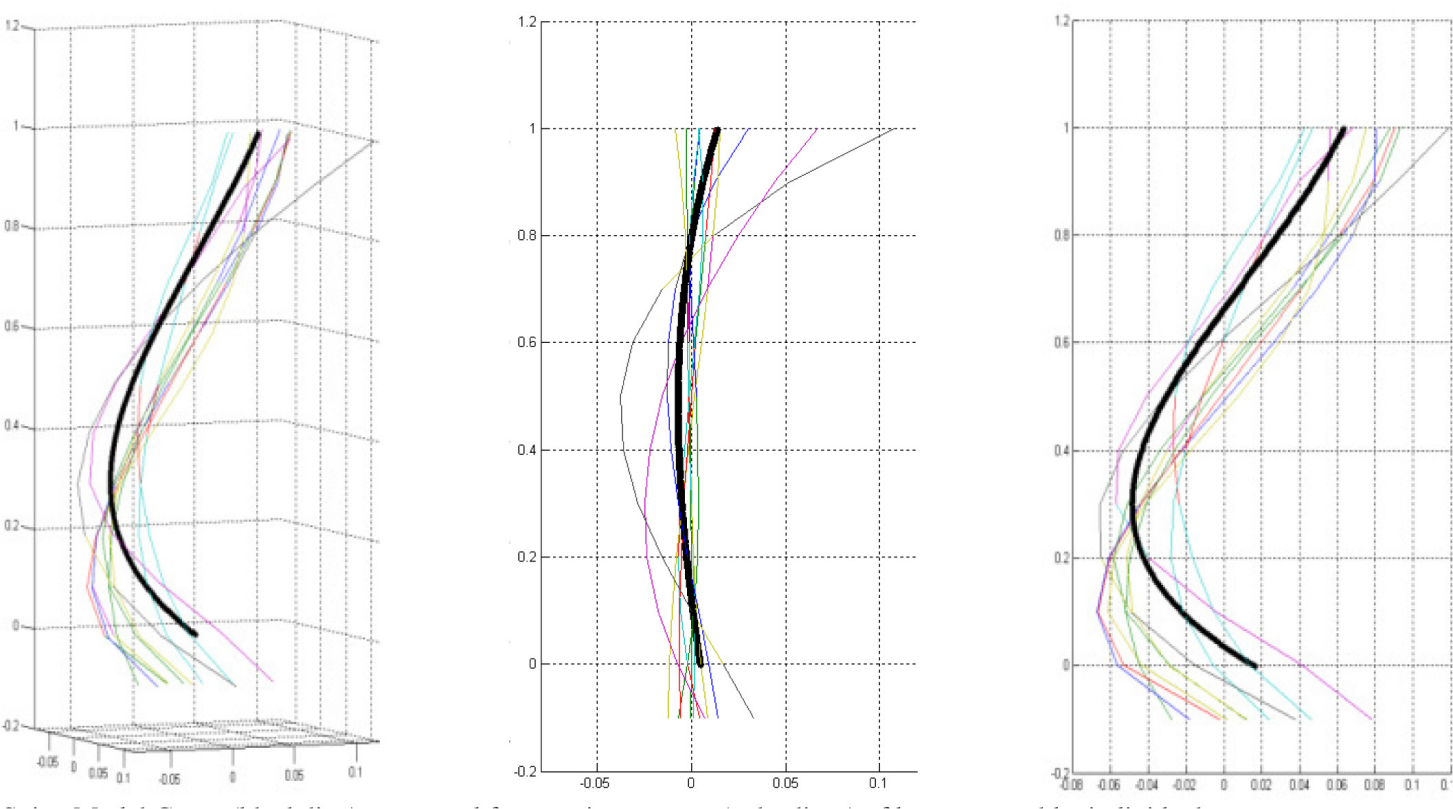
Spine Model Curve. Spine Model Curve (black line) is composed from various curves (colored lines) of healthy individuals shown in 3 different views: a 3D view (left), coronal plane (center), and the sagittal plane (right). As described, the coordinates are scaled by the vertical distance between the superior end plate of the sacrum and the inferior end plate of T12.

The modeling was formed from a sample of curves from the normal population, preprocessed as mentioned above (scaled and oriented). All curves for the model were dimensionless, oriented along a common line, had a cranial point (the intersection point of a line running parallel to the inferior discal surface of T-12 and the centerline) and a caudal common point (the intersection of a line running parallel to the superior discal surface of S-1 and the centerline) (Fig 3). A model curve was created as the median of location of all sample curves on cross-sections along the curves. The model curve’s standard deviation was the calculated standard deviation of location of all sample curves from the model curve (black and blue lines in Fig 4). For each curve, six features were measured.

**Segment Length (mm)**—the straight distance between the curve’s end points (A and B, Fig 3).

**Curve Length (mm)**—the distance along the curve between the curve’s end points (A and B, Fig 3) calculated as the sum of distances between sample points along the curve.

**Curvedness**–the difference between the lumbar curve length and its projection length (a horizontal line from caudal to cranial curve endings), calculated by dividing the curve length by projection length (the horizontal distance between the curve endings: Az-Bz, Fig 3).

**Lordosis peak location**—the position of the most ventral point on the curve, along its Z (cranial-caudal) axis. For each curve, the most ventral point along the curve was found [where the Y coordinate was maximal (point P, Fig 3) and its location measured as the horizontal distance from the caudal end point (between Pz-Bz, Fig 3)].

**Lordosis cranial peak height**—the projected distance of the curve’s peak from the cranial endpoint (Ay-Py), measured as the vertical distance (Y-axis) between the lordosis peak and the cranial end point of the curve (Fig 3).

**Lordosis caudal peak height**—the projected distance of the curve peak from the caudal end point (By-Py), measured as the vertical distance (Y-axis) between the lordosis peak and the caudal end point of the curve (Fig 3). As the B point (the intersection of a line running parallel to the superior discal surface of S-1 and the centerline) was utilized as the origin point for all lines, lumbar lordosis is defined by the amount of inward curving of the lumbar spine from this point (By-Py).

All size measurements were scaled by the projected cranial-caudal axis (Az-Bz) distance between the curve endings to produce dimensionless values.

#### Comparing curve models

In order to compare the two models i.e., revealing the differences between the 3-D graphs obtained from the males and females samples, we partitioned each graph into 20 points (equally spaced along the Z coordinate) and compared the differences between corresponding points–i.e., we examined if the difference in location of a pair sample points from the two models is statistically significant.

#### Statistical analysis

A two-tailed t-test was employed to compare continuous variables between groups. The level of significance was set at *p*< 0.05. Since the X and Y coordinates of the sample points along the curve were strongly dependent variables, a multiple statistical test correction for the curve comparison (e.g., Bonferroni [17]) was not applied to this data. The Bonferroni correction should only be used in cases where the number of tests is quite small and the correlations among the test statistics are quite low [18][18]. In the current study, correction for strongly dependent variables is required. Nonetheless, this type of correction is very small but complex and computer intensive [18] and therefore was not applied. Statistical analysis was carried out using SPSS version 14, and Matlab version 2010b.

## Results

We present herein, a description and characterization of lumbar curvature for males and females, followed by the results of the model comparisons (Table 1).

**Table 1:** Comparison of lordosis curve parameters between males and females (two-tailed t-test).

Feature	Males (N = 81)				Females (N = 77)				P value
Curve parameters	Mean	SD	Min	Max	Mean	SD	Min	Max	
**Segment Length (mm)**	**191**	**15**	**142**	**247**	**179**	**13**	**151**	**227**	**<0.01**
**Curve Length (mm)**	**194**	**15**	**144**	**251**	**182**	**13**	**156**	**228**	**<0.01**
Curvedness*	**1.019**	**0.015**	**1.000**	**1.097**	**1.023**	**0.027**	**1.001**	**1.100**	**0.049**
Lordosis peak location*	**0.299**	**0.086**	**0.120**	**0.543**	**0.274**	**0.075**	**0.120**	**0.493**	**0.032**
Lordosis cranial peak height*	**0.074**	**0.024**	**0.017**	**0.126**	**0.092**	**0.027**	**0.023**	**0.148**	**<0.01**
Lordosis caudal peak height*	**0.056**	**0.025**	**0.012**	**0.124**	**0.061**	**0.026**	**0.012**	**0.126**	**0.310**

*Scaled values

### Male population model

#### The model group

The model consisted of 81 subjects whose lumbar spine curve was extracted. The average age was 41.5±15.8 years; the youngest 18 years old, the oldest 84.

#### Curve Shape

**Fig 5:**
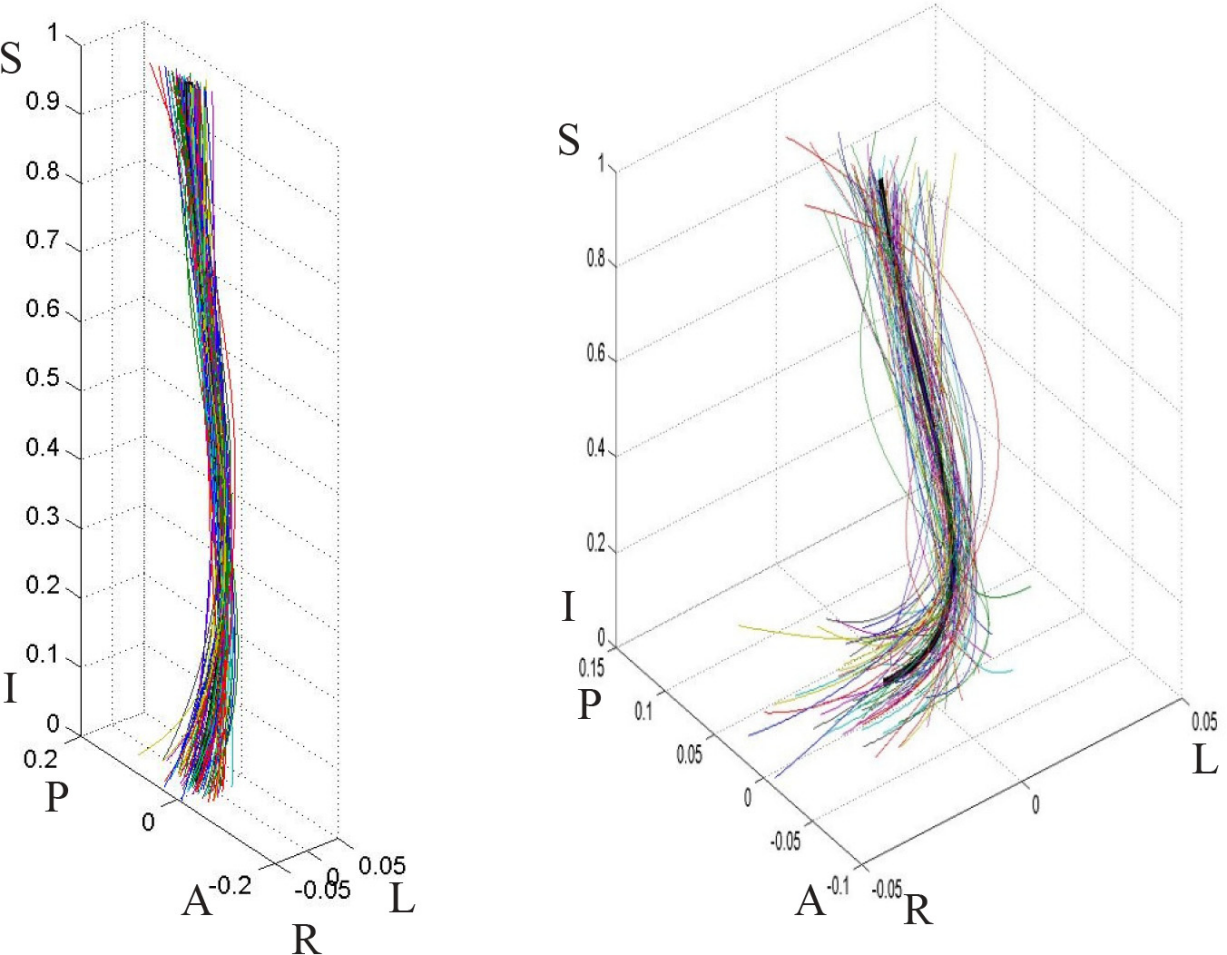
Male population curves, 3-D view. The graph on the left is scaled to the axes proportions; the graph on the right is freely scaled to arbitrary proportions for a better view of the curve shape. Each line represents a curve sample. The solid black line in the middle is the model curve. The coordinates are scaled by the vertical distance between the superior end plate of the sacrum and the inferior end plate of T12.

**Fig 6:**
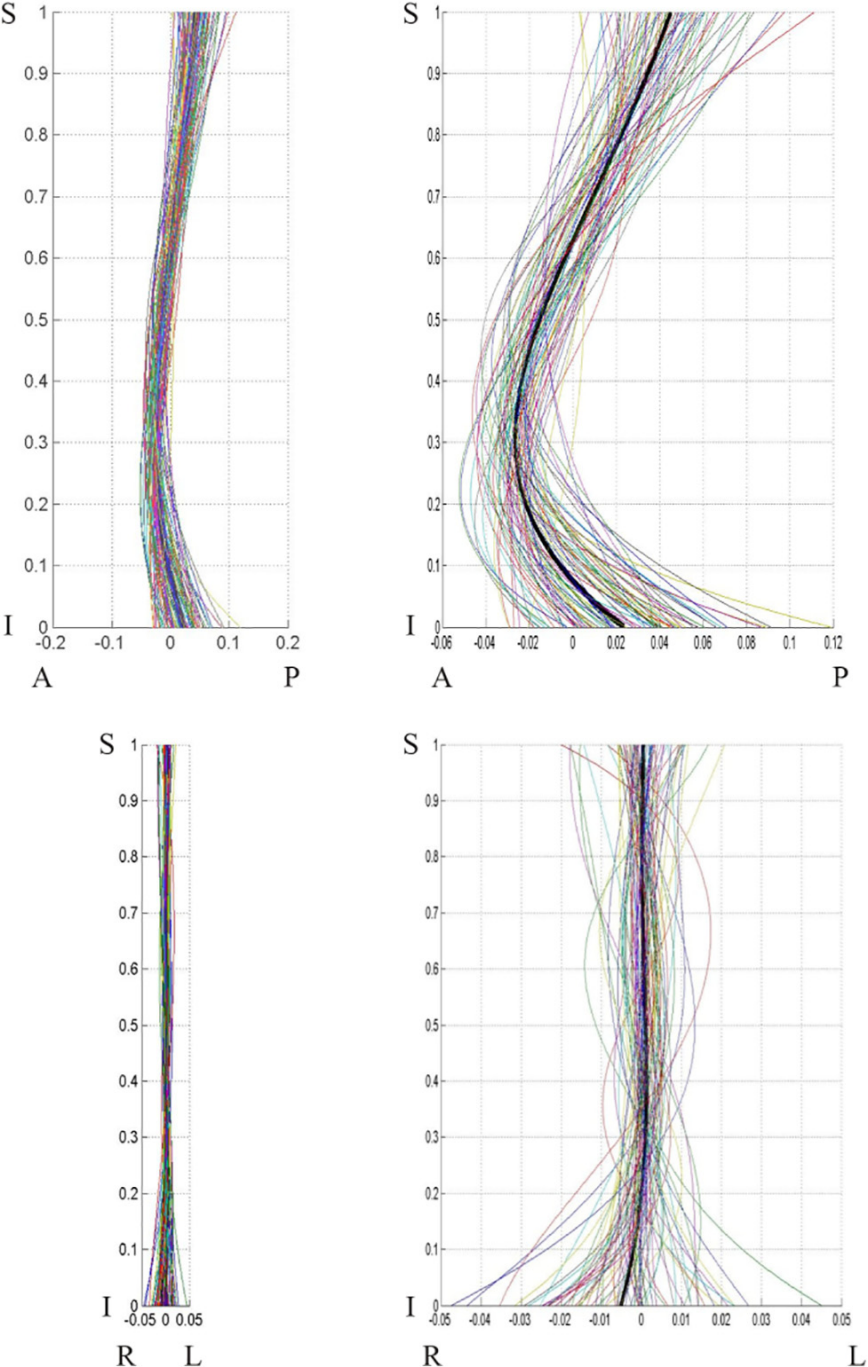
Males population curve 2-D view. Male population curve 2-D views: sagittal (top) and coronal (bottom) planes. The coordinates are scaled by the vertical distance between the superior end plate of the sacrum and the inferior end plate of T12.

The male curve shape can be seen in 3-D (Fig 5) and 2D (Fig 6). The model line shows the lordotic shape of the lumbar spine, as well as a normal minor right spinal scoliosis (Fig 6 bottom).

#### Curve Features

The results for the male curve (segment length, curve length, curvedness, lordosis peak location, lordosis cranial peak height, lordosis caudal peak height) are summarized in Table 1. Average lordosis curve length among males was 19.4±1.5 cm (range 14–25 cm). The spinal lordosis formed a rather shallow curve due to its circular shape: curvedness was 1.02 and curve peak height only 5.8% of the segment length (meaning a curve height of 11.5mm for a segment length of 191mm) when measured from the caudal end point (Fig 3, point B) and 7.4% when measured from the cranial (Fig 3, point A) end point. The curve peak location was positioned at a little more than two thirds of the lumbar spine length (70.1%±8.6) when measured from the cranial end point (A), placing it at the level of L4, nonetheless, a large range in the lordosis peak location was found from 12% (the lower part of the curve) to 54.3% (the middle of the curve).

### Female population model

#### The model group

The female model sample consisted of 77 subjects whose lumbar spine curve was correctly extracted. The average age was 41.3± 15.5 years; the youngest 18 years old, the oldest 79.

#### Curve Shape

**Fig 7:**
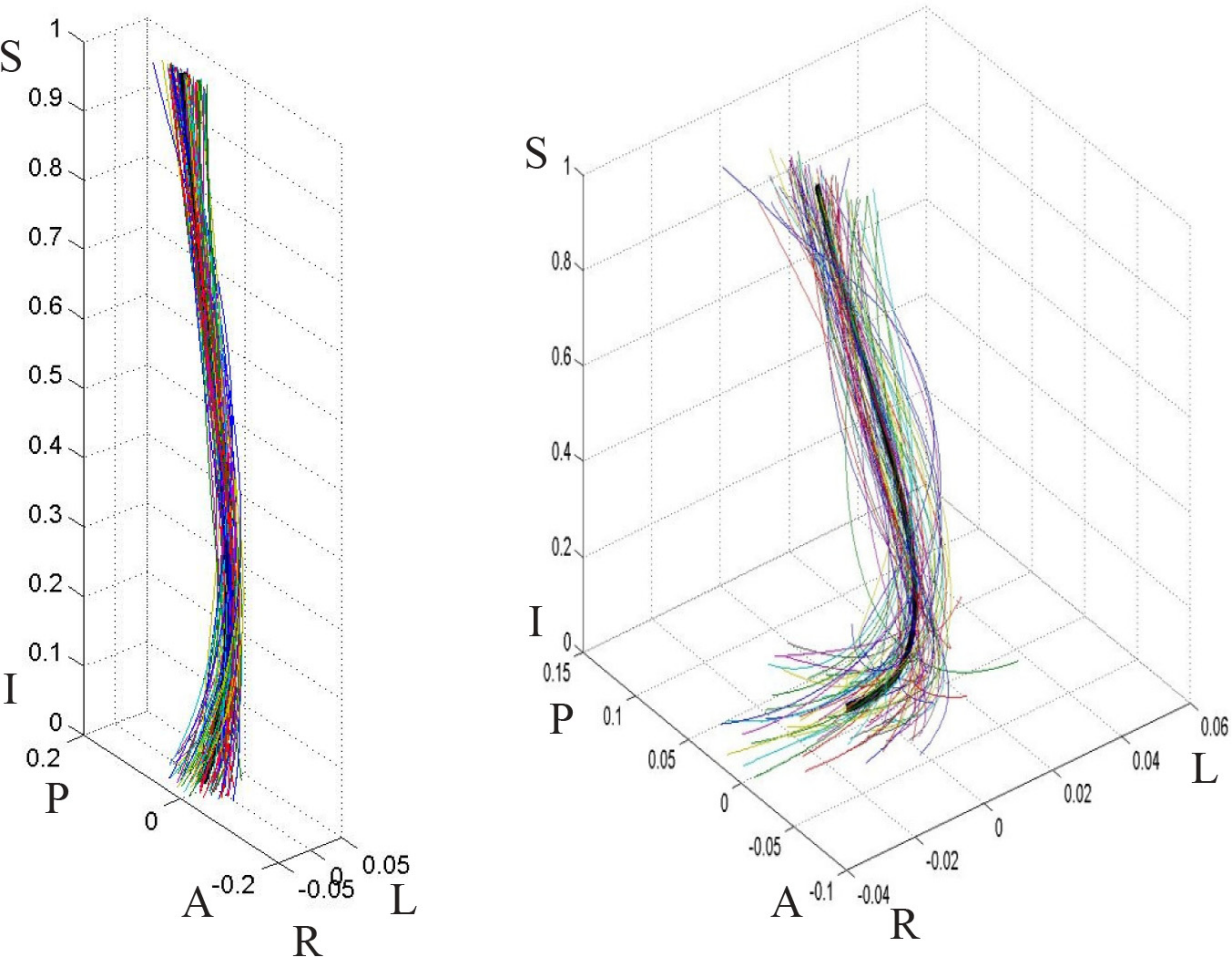
Female population curves, 3-D view. The graph on the left is scaled to the axes proportions; the graph on the right is freely scaled to arbitrary proportions for a better view of the curve shape. Each line represents a curve sample. The solid black line in the middle is the model curve. The coordinates are scaled by the vertical distance between the superior end plate of the sacrum and the inferior end plate of T12.

**Fig 8:**
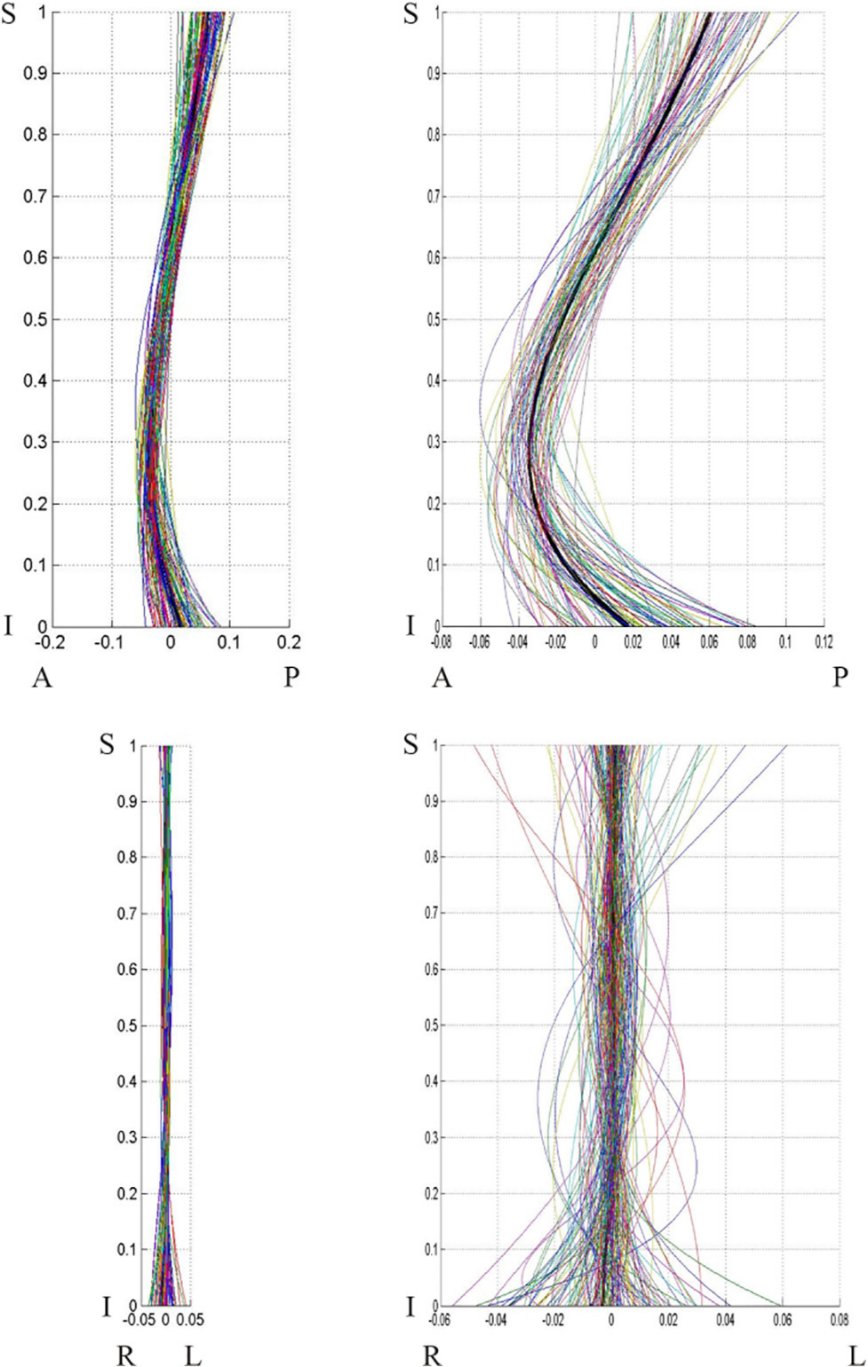
Female population curves, 2-D view. Female population curve 2-D view: sagittal (top) and coronal (bottom) planes. The graphs on the left are scaled to the axes proportions; the graphs on the right are freely scaled to arbitrary proportions for a better view of the curve shape. Each line represents a curve sample. The coordinates are scaled by the vertical distance between the superior end plate of the sacrum and the inferior end plate of T12.

The curve shapes can be seen in 3-D (Fig 7) and 2-D (Fig 8). The graphs clearly show lumbar spinal lordosis on the 3-D and 2-D sagittal graphs (Fig 8), as well as a noticeable normal minor right spinal scoliosis on the coronal graph.

#### Curve Features

The results for the female curves (segment length, curve length, curvedness, lordosis peak location, lordosis cranial peak height, lordosis caudal peak height) are summarized in Table 1. The average lordotic curve length among females was 182±13 cm (range 15–23 cm). The spinal lordosis formed a rather shallow curve due to its circular shape: curvedness was 1.023 and curve peak height was only 6.1% of the segment length (meaning a curve height of 10.9 mm for a segment length of 179 mm) when measured from the caudal end point (Fig 3, point B), and 9.1% (16.3 mm) when measured from the cranial (Fig 3, point A) end point. The curve peak location was positioned at a little more than two thirds of the lumbar spine length (72.6%±7.5) when measured from the cranial end point (Fig 3, point A)—at the level of L4. Nonetheless, a large range in the lordosis peak location was found (from 12% to 49.3%).

### Comparison of male and female models

The model curves and curve characteristics of the male and female populations are compared and described below:

#### Curve Parametric Characteristics

A comparison of lordosis curve parameters of males and females (two-tailed t-test) is presented in Table 1. Naturally, curve length was significantly greater in males than females. Female lordosis was significantly more curved than male lordosis (greater curvedness and cranial peak height). The location of the peak of the curve was significantly lower for females compared to males. It is noteworthy that caudal peak height was similar for males and females.

#### General Curve Comparison

We compared the lordosis curve shapes of males and females using the 20 location method—the distances between the lumbar curves were compared in 20 locations along the curve (Tables 2 and 3). The most caudal point (Fig 3, point B) was dropped, since it was similar for all lines (defined as the origin). The results were divided into curve location in Y (dorsal-ventral) (Table 2) and curve location in X (lateral-lateral) (Table 3).

**Table 2:** Model comparison: Comparison of curve shape on the sagittal plane (Y axis = dorsal-ventral) between males and females.

Location	Males (N = 81)		Females (N = 77)		P value
	Mean	SD	Mean	SD	
1	0.021	0.033	0.040	0.036	>0.01
2	0.016	0.033	0.033	0.035	0.001
3	0.010	0.032	0.026	0.035	0.002
4	0.004	0.033	0.019	0.035	0.004
5	-0.002	0.033	0.011	0.035	0.010
6	-0.009	0.033	0.003	0.035	0.022
7	-0.016	0.034	-0.006	0.036	0.047
8	-0.023	0.034	-0.014	0.036	0.086
9	-0.029	0.035	-0.022	0.035	0.147
10	-0.036	0.035	-0.030	0.035	0.238
11	-0.041	0.034	-0.036	0.035	0.354
12	-0.046	0.033	-0.042	0.034	0.508
13	-0.049	0.032	-0.047	0.033	0.683
14	-0.050	0.030	-0.049	0.031	0.879
15	-0.049	0.028	-0.050	0.029	0.909
16	-0.046	0.025	-0.047	0.026	0.714
17	-0.040	0.021	-0.042	0.022	0.541
18	-0.030	0.015	-0.032	0.017	0.396
19	-0.017	0.009	-0.018	0.009	0.290

**Table 3:** Model comparison: Comparison of curve shapes on the coronal plane (X-axis = lateral-lateral) between males and females.

Location	Males (N = 81)		Females (N = 77)		P value
	Mean	SD	Mean	SD	
1	0.005	0.020	0.005	0.018	0.780
2	0.004	0.019	0.005	0.018	0.750
3	0.004	0.019	0.005	0.018	0.709
4	0.004	0.019	0.005	0.019	0.669
5	0.004	0.019	0.005	0.019	0.642
6	0.004	0.019	0.005	0.020	0.631
7	0.004	0.019	0.005	0.020	0.637
8	0.004	0.020	0.005	0.020	0.661
9	0.004	0.020	0.005	0.020	0.701
10	0.005	0.020	0.005	0.020	0.761
11	0.005	0.020	0.005	0.020	0.832
12	0.005	0.019	0.005	0.020	0.918
13	0.005	0.019	0.005	0.019	0.991
14	0.005	0.018	0.005	0.018	0.899
15	0.005	0.016	0.004	0.017	0.806
16	0.004	0.015	0.004	0.015	0.724
17	0.004	0.012	0.003	0.013	0.652
18	0.003	0.009	0.002	0.010	0.590
19	0.001	0.005	0.001	0.005	0.543

**Fig 9:**
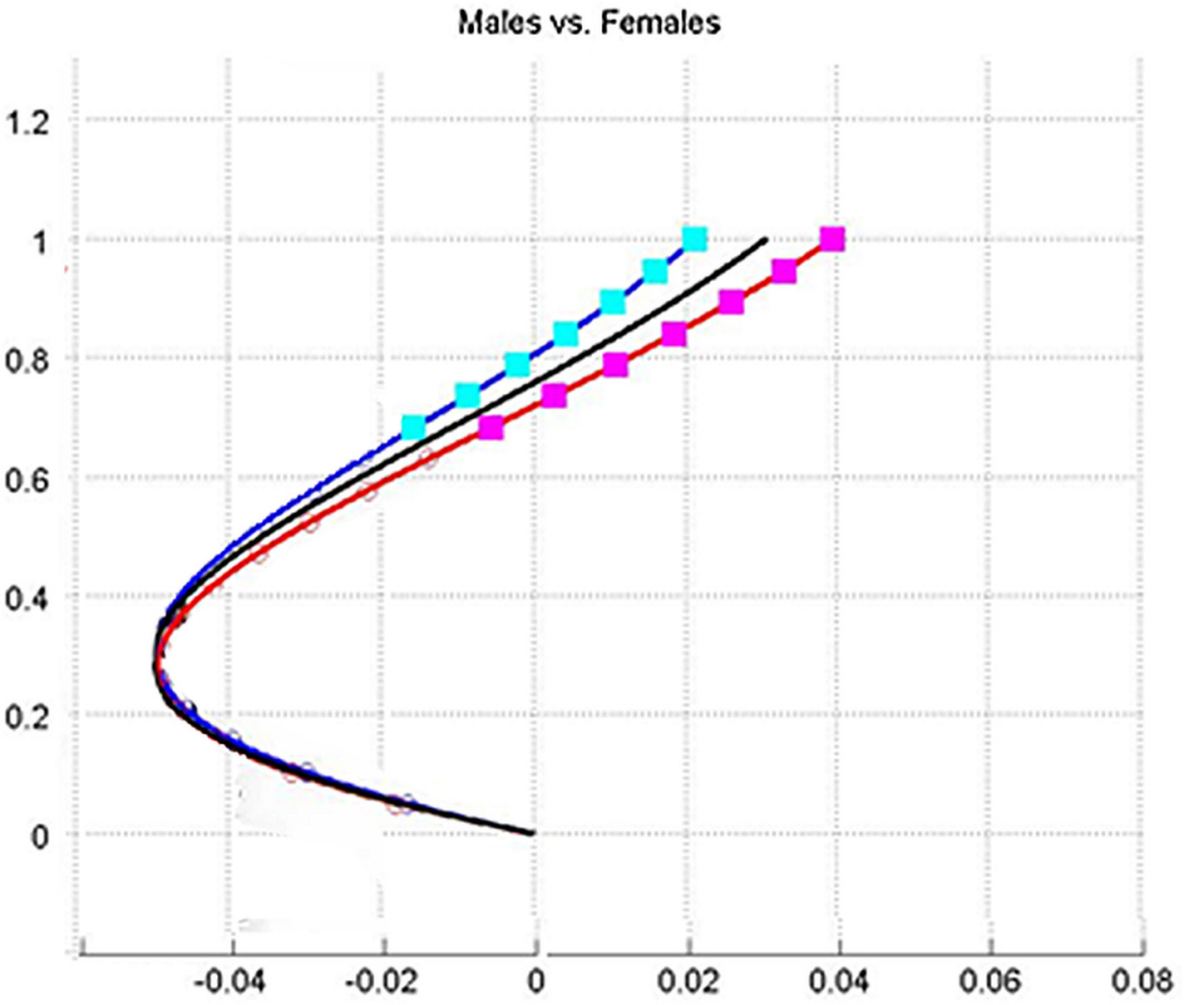
Models of male population vs. female population. Graphical comparison of the sagittal plane of the curve shape between male and female populations: black line—general population; blue line—male population; red line—female population. Squares denote locations of significant differences (p < 0.05) between the curves. The graph is scaled to arbitrary proportions for a better view of curve differences.

Significant differences between the curves on the Y-axis were obtained for most cranial points (N = 7), implying that the upper part of the lordotic curve in females is more dorsally placed (backwardly inclined) relative to a vertical line passing through the caudal common point) compared to males. This is clearly visible on the graph (Fig 9). On the X-axis, no significant differences were found between males and females.

## Discussion

Our results show that the shape of the female lumbar curve differs from that of the male in three aspects: greater curvature (the curve is more pronounced), the upper segment is located more dorsally (backwardly inclined), and the peak of the curve is situated more caudally (inferiorly) (Fig 9). The greater curve found in females is in accordance with some previous studies [2, 9, 11], but counter to others [10] where no statistically significant differences between the sexes were found.

In the current study, the elements that compose the lumbar arch (vertebrae and discs) and how they were modified to reach the existing curve was not examined, as has been done by previous studies [19, 20]. The properties of the curve itself were examined which allowed us to rephrase the major questions associated with the male/female differences relating to the human lumbar curve, namely: why does the female lumbar curve manifest a greater curvedness and more inferior peak location compared to males? Why is the superior part of their lumbar spine curve positioned more dorsally (backwardly)?

Both female and male lumbar curves are designed to ensure stability when standing and walking, yet spinal configuration in females must take into account potential abrupt periodical changes (mainly during and shortly after pregnancy) in their center of mass (COM). An increase of lumbar lordosis during pregnancy, especially during the last months is well documented [19, 21, 22]. Furthermore, it has been shown that the degree of lumbar lordosis in women is significantly associated with the number of pregnancies [22]. Whitcome et al. [21] stated that during pregnancy, women naturally increased their lumbar lordosis in order to retain their relatively stable COM, by no more than 1cm. The observed configuration of the spine as seen in our study suggests that there is a-priori adaptation to minimize forward translation of COM during pregnancy, without which further increase in lumbar lordosis would have been required. The postural alignment of the female spine (i.e., curve peak located more caudally in females, and the more backwardly inclined thoracolumbar area) minimizes the need for additional lumbar lordosis during pregnancy [21].

Despite extensive research on the relationship between lumbar lordosis and pregnancy, the reasons behind these relationships are not fully understood. Some have suggested that increased lumbar lordosis provides females with a larger space for the fetus to develop, especially when the baby is forced into a flexed position due to space limitation [19]. Many authors have claimed that the increase of lordosis among females compensates for the increase of body weight during pregnancy, followed by the change in the torso center of mass [21–25].

Moreover, a prominent anatomical difference between males and females is the presence of enlarged external breasts suspended over the chest. This difference may be even more significant during pregnancy and afterwards, during lactation, when breast size may double. These anatomical changes may cause forward position of the COM, which in turn affects spine stability when standing and walking. In order to compensate for this potential instability, a larger thoracic kyphosis is required. Curve change at the thoracolumbar spine from lordosis to kyphosis is taking place over several motion segments to avoid abrupt change in the angulations of adjacent vertebrae at a specific area of the thoracolumbar spine. Lower lordosis peak allows more motion segments that can take part in this change. This may explain why the curve’s peak location in females must be situated lower than the males. A larger thoracic kyphosis generates enlargement of the lumbar lordosis in order to increase the posture’s biomechanical stability [26].

Whitcome et al. [21] stated that females present a longer series of dorsally wedged lumbar vertebrae than males, with relatively larger and more frontally oriented facets, presenting greater resistance to forward displacement of the lumbar vertebrae during pregnancy. Masharawi et al. [19, 27] suggested that the combination of a smaller kyphotic vertebral body wedging into the lower thoracic and upper lumbar vertebrae, the relatively greater interspinous spacing, and the larger interfacet width in the lumbar spine in females, are key architectural elements in the female’s spinal adaptation to pregnancy. However, a word of cautious is needed, in both Whitcome et al. [21] and Masharawi et al. [19, 27] studies, it is not clear whether the above described bone modifications equal real differences in lordosis between the two sexes.

The advantage of a greater lumbar curve and especially the more dorsal (backward) placement of the upper curve for females is their ability to more easily counter act the forward placement of the center of gravity during pregnancy, thus reducing the mechanical load on the lumbar region [28, 29]. Merely increasing the lumbar curve would exposes the lumbar extensor muscles to increased stress [30] and would poses a higher shear load on the lumbar neural arches.

The effects of a greater lumbar lordosis with regard to pregnancy, is an increase in spinal anomalies such as degenerative spondylolisthesis, four times more common in females [31] and two times higher in multiparous females than nulliparous [32], a decrease in rotational stability (which suggests why idiopathic scoliosis occurs more frequently in females [33]), and higher prevalence of low back pain due to the narrowing of the intervertebral foramen in hyperextension.

The transition to a vertically oriented spine from a semi-orthograde spinal orientation during human evolution resulted in the gravity vector considerably realigning from its original axis, a change that has led to dramatic kinetic and kinematic changes in the human spine. Balancing the torso over the hips, especially in pregnant women during the single limb support phase, became an issue. The unique series of alternating curvatures that characterize the human spine (as shown here) allows the torso to balance more efficiently over the extended lower limbs. The human spine (relative to the great apes) has invaginated ventrally (forward) into the abdominal cavity in order to closely coincide the center of mass with the vertebrae centers of rotation, thereby reducing torsional and bending stresses on the spine and increasing the mechanical moment produced by the erector spinae muscles [34], thus also assisting in balancing the torso over the interacetabular axis during pregnancy.

This biomechanical scenario is supported by our findings that caudal peak height is similar for males and females. In turn, this implies the existence of mechanical constraint, as a more ventrally placed peak would move the line of gravity too far forward, resulting in balancing difficulties of the torso over the lower limbs, and would therefore greatly hamper efficient posture (in terms of energy cost) and bipedal locomotion. Increase mass in the anterior trunk, i.e., pregnancy, nursing and carrying babies, therefore, may cause these problems to intensify.

Finally, two issues regarding the validity of the above observations may be raised: it may be argued that the positional alignment of the body during data acquisition (e.g., subject standing or lying supine on a bed, subject with legs flexed or extended, subject with or without cochin support to the lower back) and the extent of soft tissue support under the body (differences in the gluteus maximus cross-sectional area and dorsal thoracic muscle mass) might lead to diverse curvedness among individuals and between sexes.

In our study, however, all patients were scanned in the same standard manner. The differences in soft tissue mass between males and females may have only a minimal effect on the spinal canal alignment as the construction of the curve is independent of the distance between the vertebrae and the bed. The major obstacle could have been the position of the body, yet it has been demonstrated in several papers that the lordotic curve does not significantly change when measured in an upright or horizontal position [34, 35].

The second issue relates to the wide age range of participants in this study. Indeed, it is well known that the shape of the spine changes with age and that the dynamics of the change may differ between the sexes. In the current paper, however, we deemed it appropriate and more beneficial to compare the entire spectrum of spinal shapes between males and females which is why we presented individual curves, not just the averages (Fig 9). By doing so, we simplified the comparison of lumbar curves between the sexes without losing the extent of variation throughout the ages.

## Conclusions

There is a fundamental difference in spinal lumbar curve shape between males and females: the female spine manifested a greater curvature, a caudally located lordotic peak, and greater cranial peak height. Nevertheless, amount of inward curving (lordosis) is sex-independent. The architectural differences in lumbar lordosis shape between the sexes are due to dissimilarity in the conditions and constraints under which the male and female spines operate.
